# A Case of Novel Corona Virus With Three Negative Nasopharyngeal Swabbings

**DOI:** 10.7759/cureus.8266

**Published:** 2020-05-24

**Authors:** Tianyu She, Haowei Han, Sandeep Gandhi

**Affiliations:** 1 Internal Medicine, New York College of Osteopathic Medicine, Old Westbury, USA; 2 Family Practice, Peconic Bay Medical Center-Northwell Health, Riverhead, USA; 3 Medicine, Peconic Bay Medical Center-Northwell Health, Riverhead, USA

**Keywords:** covid-19, rt-pcr, diagnostic testing, false-negative, medical screening

## Abstract

We hereby report a case of a 55-year-old male with fever and difficulty breathing over several days who treated for presumed COVID-19 pneumonia despite testing negative thrice via reverse transcription polymerase chain reaction (RT-PCR) nasal swab. We explore several possible reasons for serially negative SARS-CoV-2 testing and other potential avenues of diagnosis.

## Introduction

In the past 20 years, there have been three severe coronavirus outbreaks. In 2002, there was a worldwide outbreak of severe acute respiratory syndrome (SARS-CoV). In 2012, there was an outbreak of Middle East Respiratory Syndrome (MERS-CoV). At the end of 2019, a novel coronavirus (SARS-CoV-2) was identified as being responsible for a cluster of viral pneumonias in Wuhan, China [1].

Since the first confirmed novel coronavirus (SARS-CoV-2) virus infection in Washington state on January 30th, 2020, the prevalence of COVID-19 pneumonia in the United States has rapidly exceeded other countries [2,3]. The diagnosis relies on real-time reverse transcription polymerase chain reaction (RT-PCR) utilizing nasopharyngeal or oropharyngeal samples. To the authors’ knowledge, there was no established research about the predictive values of RT-PCR [2,4]. Meanwhile, the Center for Disease Control and Prevention has developed a novel serology testing for SARS-CoV-2. Nonetheless, the timeframe from SARS-CoV-2 exposure to the production of detectable serum antibodies is one to two weeks, limiting serology testing as diagnostic in the acute setting [4].

## Case presentation

**Figure 1:**
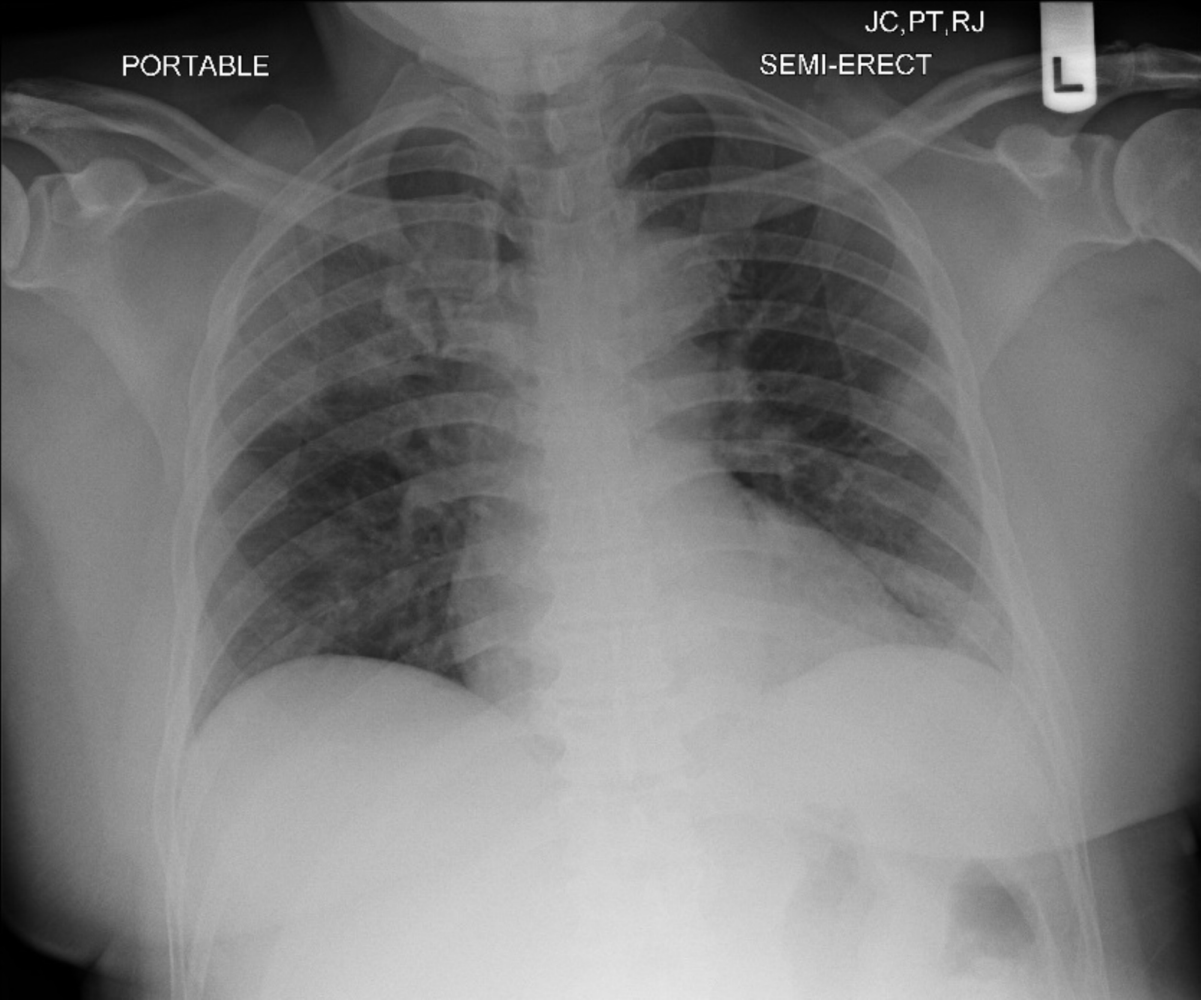
Chest X-ray (day 1) revealed right upper lobe and bilateral lower lobe infiltrates

**Figure 2:**
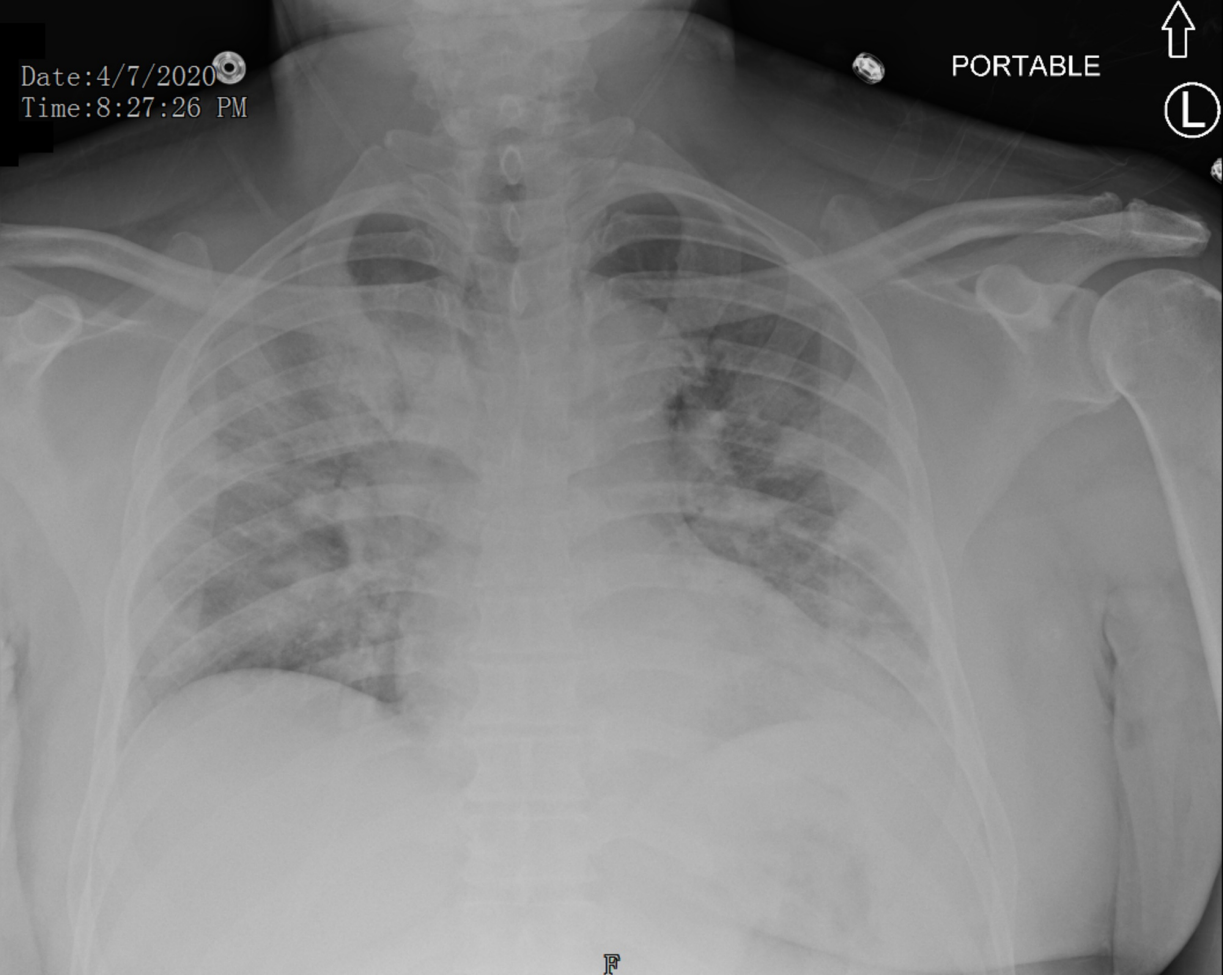
Chest X-ray (day 2) revealed bilateral infiltrates

A 55-year-old African American male with past medical history of hypertension, diabetes, and hyperlipidemia was admitted for fever and difficulty breathing for several days. His associated symptoms were chest tightness without any pain. A diagnosis of possible community-acquired coronavirus was made. His vital signs were a temperature of 101.8 °F, heart rate of 100 beats/min, blood pressure of 163/101 mmHg, respiratory rate of 16 breaths/min, and pulse oximetry of 96% on room air. Physical examination was remarkable for decreased breath sounds without any rales or rhonchi. The patient was placed on nasal cannula. Tests for influenza and respiratory syncytial virus by PCR were negative. Initial chest X-ray (CXR) revealed right upper lobe and bilateral lower lobe infiltrates (Figure 1), without hilar fullness, congestion, or Kerley lines. Complete blood count and complete metabolic panel were remarkable for sodium of 133 mmol/L, blood glucose of 230 mg/dL, and a creatinine of 1.51 mg/dL. Troponin was elevated at 0.307 ng/ml and it slowly trended down afterwards. He was transferred to our facility on hospital day 2. The initial workup at our facility was unremarkable with a creatinine of 1.2 mg/dL and negative troponin level. Hemoglobin A1c was 8.7%. His inflammatory markers were markedly elevated, with a C-reactive protein (CRP) of 34.86 mg/dL and a D-dimer of 462. A CXR showed bilateral infiltrate (Figure 2). He was placed on airborne and contact precautions. He was started on oral hydroxychloroquine and intravenous ceftriaxone with the eventual addition of doxycycline for a suspected superimposed bacterial pneumonia. His home medication of Losartan was held during hospitalization. Repeated nasal COVID PCR swabs on hospital day 2 and 3 were negative. Blood and urine cultures were negative as well. Electrocardiogram revealed left bundle branch block. Echocardiogram suggested by cardiologist revealed a reduced ejection fraction of 35%. The patient was placed on 4-5 liters of oxygen via nasal cannula throughout the hospital course and his symptoms were well controlled. Subsequently, he was able to wean off the supplemental oxygen. His CRP and D-dimer trended down. He was discharged with instructions on self-isolation.

## Discussion

Like the previous two coronaviruses, there is no specific clinical features that can allow clinicians to distinguish SARS-CoV-2 from other severe viral illnesses. From an epidemiological study involving 1099 patients with laboratory-confirmed positive SARS-CoV-2 testing, the most common clinical features at the onset of illness were fever, which was present in 43.8% on admission and 88.7% during hospitalization, and a cough (67.8%). Other laboratory findings included lymphocytopenia (83.2%), thrombocytopenia (36.2%), elevated CRP (60.7%), elevated lactate dehydrogenase (41.0%), transaminitis (aspartate aminotransferase 22.2% and alanine aminotransferase 21.3%), and an elevated D-Dimer (46.4%) [5].

Currently, the U.S. Food and Drug Administration has approved widespread testing for SARS-CoV-2 by real-time polymerase chain reaction [6]. Our patient was tested negative three times with nasopharyngeal swabbings and subsequent RT-PCR testing. Initial false-negative RT-PCR testing is not uncommon. This is possibly due to the patient’s early disease course with mild symptoms. One study involving 76 patients in Nanchang, China, found that the mean viral load of severe cases was around 60 times higher than that of mild cases, which suggests that higher viral loads are correlated with more severe cases [7]. Our patient had only mild symptoms with fever and shortness of breath at his initial presentation and he only needed oxygen via nasal cannula. In another retrospective cohort study by Northwell Heath facilities in New York, only 13/5600 patients (2%) had a negative first test and positive repeat test [2]. In addition, our patient was started on hydroxychloroquine with a presumed diagnosis of COVID-19 pneumonia. A non-randomized small population clinical trials have shown that hydroxychloroquine treatment is significantly associated with decreased viral load, possibly further contributing to subsequent negative RT-PCR testing [8].

Another probable explanation is seen in the entry pattern SARS-CoV-2. Recent literature has suggested that the novel coronavirus uses the same cell entry receptor, angiotensin-converting enzyme II (ACE2) as SARS-CoV [9]. Literature from 2004 identified that type II pneumocytes express the ACE2 protein, and that tissues of the upper respiratory tract, including the nasopharynx, oral and nasal mucosa, did not [10]. This may possibly explain why, despite the small sample size, the highest rates of positive viral RNA findings at 93% in 15 samples were reported from bronchoalveolar lavages versus 72% in 104 sputum samples, 63% in eight nasal swab samples, and 32% in 398 oropharyngeal swab samples [11].

Other avenues should be investigated to aid in the diagnosis of SARS-CoV-2. CXR in our patient showed bilateral airspace disease consistent with viral pneumonia. One study showed that CXR findings were similar to those found in CT, which demonstrate bilateral peripheral consolidation. CXR had a lower sensitivity than initial RT-PCR testing, with 69% versus 91%, respectively, with the referenced gold standard being an eventual positive RT-PCR [12]. CT imaging was not done on our patient due to fear of contamination and spread; however, perhaps in the future, it may be utilized as a reliable alternative to RT-PCR testing. In a retrospective study looking at 121 CT scans of symptomatic COVID-19 patients, they determined that the hallmark of COVID-19 infection is a pattern of ground-glass and consolidative pulmonary opacities, with a bilateral and peripheral lung distribution [13]. With the development of rapid detection with a high sensitivity rate, infection and spread can be contained appropriately. In a study involving 1014 patients with eventual positive RT-PCR testing as the reference, 60% to 93% of patients had initial positive chest CT consistent with COVID-19 before the initial positive RT-PCR testing results [14].

With RT-PCR testing as the current gold-standard test for our overburdened healthcare system, we must carefully evaluate its role in guiding decisions of management. Looking at SARS-CoV, which has a matching genetic sequence of over 70% to COVID-19, detectibility of RT-PCR peaks nine to 11 days after onset of illness, making RT-PCR less useful during the first week of illness [15,16]. Similarly, in a study that included 75 patients, 24 (32%) had a positive RT-PCR at the initial presentation compared to 51 (86%) on day 14 [17].

## Conclusions

Learning from the past, we must clinically assess the patient holistically, weighing in factors of etiological testing, imaging, travel, contacts, comorbidities, and location of sampling, and possibly treating and isolating patients for COVID-19 even with serially negative RT-PCR testing. In addition, more studies addressing the predictive values of RT-PCR, and other effective diagnostic methods should be conducted.

## References

[1] Wang C, Horby P, Hayden F, Gao G (2020). A novel coronavirus outbreak of global health concern. Lancet.

[2] Holshue ML, DeBolt C, Lindquist S (2020). First case of 2019 novel coronavirus in the United States. N Engl J Med.

[3] Richardson S, Hirsch J, Narasimhan M (2020). Presenting characteristics, comorbidities, and outcomes among 5700 patients hospitalized with COVID-19 in the New York City area. JAMA.

[4] (2020). Information for laboratories about coronavirus (COVID-19). https://www.cdc.gov/coronavirus/2019-ncov/lab/index.html.

[5] Guan W, Ni Z, Hu Y (2020). Clinical characteristics of coronavirus disease 2019 in China. N Engl J Med.

[6] (2020). FAQs on testing for SARS-CoV-2. https://www.fda.gov/medical-devices/emergency-situations-medical-devices/faqs-diagnostic-testing-sars-cov-2.

[7] Liu Y, Yan L, Wan L (2020). Viral dynamics in mild and severe cases of COVID-19. Lancet.

[8] Gautret P, Lagier JC, Parola P (2020). Hydroxychloroquine and azithromycin as a treatment of COVID- 19: results of an open-label non-randomized clinical trial. Int J Antimicrob Agents.

[9] Zhou P, Yang X, Wang X (2020). A pneumonia outbreak associated with a new coronavirus of probable bat origin. Nature.

[10] Hamming I, Timens W, Bulthuis ML, Lely AT, Navis GJ, van Goor H (2004). Tissue distribution of ACE2 protein, the functional receptor for SARS coronavirus. A first step in understanding SARS pathogenesis. J Pathol.

[11] Wang W, Xu Y, Gao R, Lu R, Han K, Wu G, Tan W (2020). Detection of SARS-CoV-2 in different types of clinical specimens. JAMA.

[12] Wong H, Lam H, Fong A (2019). Frequency and distribution of chest radiographic findings in COVID-19 positive patients. Radiology.

[13] Bernheim A, Mei X, Huang M (2020). Chest CT findings in coronavirus disease-19 (COVID- 19): relationship to duration of infection. Radiology.

[14] Ai T, Yang Z, Hou H (2020). Correlation of chest CT and RT-PCR testing in coronavirus disease 2019 (COVID-19) in China: a report of 1014 cases. Radiology.

[15] Hui D, Azhar E, Madani T (2020). The continuing 2019-NCoV epidemic threat of novel coronaviruses to global health — The latest 2019 novel coronavirus outbreak in Wuhan, China. Int J Infect Dis.

[16] Tang P, Louie M, Richardson SE (2004). Interpretation of diagnostic laboratory tests for severe acute respiratory syndrome: the Toronto experience. CMAJ.

[17] Peiris JSM, Chu CM, Cheng VVC (2003). Clinical progression and viral load in a community outbreak of coronavirus-associated SARS pneumonia: a prospective study. Lancet.

